# Retraction: MicroRNA-573 inhibits cell proliferation, migration and invasion and is downregulated by PICSAR in cutaneous squamous cell carcinoma

**DOI:** 10.17305/bjbms.2022.8232

**Published:** 2023-01-06

**Authors:** BJBMS Editors

Retraction: MicroRNA-573 functions as a tumor suppressor and is downregulated by PICSAR in cutaneous squamous cell carcinoma [1]. This article has been withdrawn at the request of the authors. The authors voluntarily requested the retraction of this paper. The additional experiments they conducted could not confirm the role of microRNA-573 functions in cutaneous squamous cell carcinoma reported in the BJBMS article (vol. 22(2):229–237). All authors signed and agreed on the retraction of the article. The Bosnian Journal of Basic Medical Sciences apologizes to the readers and scientific community for the inconveniences caused by this retraction.
